# Asymptomatic nonossifying fibroma-like lesions in neurofibromatosis type 1 patients

**DOI:** 10.1302/2633-1462.78.BJO-2026-0044.R1

**Published:** 2026-08-20

**Authors:** Maciej J. K. Simon, Johannes Salamon, Said Farschtschi, Lan Kluwe, Annika Erdmann, Victor Mautner, Andreas Niemeier

**Affiliations:** 1 Department of Orthopaedics and Trauma Surgery, University Medical Center Schleswig-Holstein – Campus Kiel, Kiel, Germany; 2 Department of Orthopaedics, University Medical Center Hamburg-Eppendorf, Hamburg, Germany; 3 Department of Diagnostic and Interventional Radiology, University Medical Center Hamburg-Eppendorf, Hamburg, Germany; 4 Department of Neurology, University Hospital Hamburg-Eppendorf, Hamburg, Germany; 5 Department of Orthopaedics and Traumatology, Reinbek Hospital, Reinbek, Germany

**Keywords:** Neurofibromatosis type 1, MRI, Non-ossifying fibroma, NOF-like lesions, lesions, neurofibromatosis type 1, MR-imaging, orthopaedic surgeon, fibrous dysplasia, physicians, skeleton, femur, tibia, bony lesions

## Abstract

**Aims:**

Neurofibromatosis type 1 (NF1) is genetic syndrome involving multiple tissues and organs including the skeleton. Because of prevalent tumours, NF1 patients often receive an MRI scan for the whole body. On MRI, skeletal findings resembling nonossifying fibroma (NOF) are frequently found. The clinical relevance of these lesions is unknown, so that diagnosis often leads to uncertainty for patients and physicians. The present study aims to explore the frequency of MRI-detectable NOF-like lesions and retrospectively evaluate associated clinical symptoms.

**Methods:**

A total of 333 NF1 patients who received at least one whole-body MRI were included. These MRI scans were assessed by a radiologist and an orthopaedic surgeon for NOF-like signals. For patients with NOF-like findings, medical records were re-evaluated to identify associated clinical symptoms.

**Results:**

A total of 127 NOF-like lesions were observed in 63 out of the 333 patients (19%). The most frequent region of the radiological lesions was the femur (57%), followed by the tibia (25%). Histology of two such lesions were NOF and fibrous dysplasia. For the majority (84%) of the patients with such NOF-like lesions, no associated symptoms were identified. Ten patients (16%) had mild transient symptoms in the proximity of their NOF-like lesions. However, extensive and follow-up examination excluded an association of these symptoms with the radiological lesions.

**Conclusion:**

NOF-like lesions are frequent on whole-body MRI scans of NF1 patients. However, there is no association with clinical symptoms. No specific treatment is indicated. These NOF-like lesions should be recognized as ‘no-touch’ lesions.

Cite this article: *Bone Jt Open* 2026;7(8):1110–1117.

## Introduction

Neurofibromatosis type 1 (NF1) is a genetic disorder with a prevalence of approximately one in 3,000 people in Germany, and is characterized by multiple tumours originating from Schwann cells of the peripheral nerves.^1^ The hallmark feature is cutaneous abnormalities, and the syndrome involves multiple organs and tissues, including the skeleton.^2^

In our specialized NF1 outpatient clinic, MRI scans are conducted for most of the patients with a confirmed diagnosis of NF1, primarily aiming for screening deeper plexiform neurofibromas which can develop in any region of the body.^3-5^ On these MRI scans, intraosseous nonossifying fibroma-like (NOF-like) signals are frequently noticed.^6,7^ In contrast to spinal deformities, congenital pseudarthrosis of the tibia, and osteoporosis, which are relatively well described skeletal abnormalities in NF1,^8-10^ little is known about the NOF-like lesions in NF1. Both physicians and families are often concerned about the potential clinical consequences. In some cases, surgery is carried out for fear of expansion of the NOF-like lesions. Biopsies are sometimes taken. However, there is no reliable evidence for the clinical relevance of these radiological NOF-like lesions so far. In addition, reports of these skeletal lesions remain anecdotal and prevalence has not been systematically assessed.

Therefore, the present study was designed to systematically identify the prevalence of NOF-like lesions on whole-body MRI scans in a unique population of over 300 NF-1 patients in an observational study over 15 years and to evaluate the clinical relevance of these lesions.

## Methods

### Study population

This is a retrospective analysis of clinical records from a period of 15 years (January 2003 to December 2018) of patients who received medical care in our Neurofibromatosis Outpatient Clinic, a specialized facility for neurofibromatosis. Patients with confirmed diagnosis of NF1 and at least one whole-body MRI scan were included.

The study was performed in accordance with the latest update of the Declaration of Helsinki.^11^ Ethical approval was obtained from the local ethics committee (registration number: PV5802). All included patients and/or their legal guardians gave written informed consent. After extracting medical findings from the charts, all data were completely anonymized. Only the results of the evaluation are presented in this report.

Age ranged from an IQR of three to 73 years (median 28.9). Approximately half of the patients were male patients (n = 170).

### Radiological assessment

MRI scans were performed on 1.5-Tesla scanners (MAGNETOM Avanto; Siemens Healthcare, Erlangen, Germany) from January 2003 to December 2011, and on 3-Tesla scanners (MAGNETOM Skyra; Siemens Healthcare) from January 2012 to December 2018. MRI scans were first evaluated for intraosseous lesions by a board-certified radiologist (JS) with training in musculoskeletal disease. MRI scans with detected and suspected NOF-like lesions were reassessed by an orthopaedic surgeon (MS, AN). For radiologically confirmed cases with NOF-like lesions, medical records of the patients were reviewed and re-evaluated for potentially associated symptoms. In addition, these NOF-like lesions were measured volumetrically in three plains by using a biologic-dynamic ellipsoid calculating formula (a × b × c × 0.523).^12,13^

NOF-like lesions were categorized by skeletal location based on the AO classification system.^14^ For 92 out of the 122 lesions, MRI at two different dates were available. Annual changes of the volume of the lesions per year were calculated as: ((volume at follow-up MRI)–(volume at initial MRI))/(years between the two MRIs).

### Histology

In two cases, because the patients and their healthcare providers were concerned for the radiologically detected NOF-like lesions, the lesions were biopsied and histologically evaluated.

### Data evaluation

**Fig. 1:**
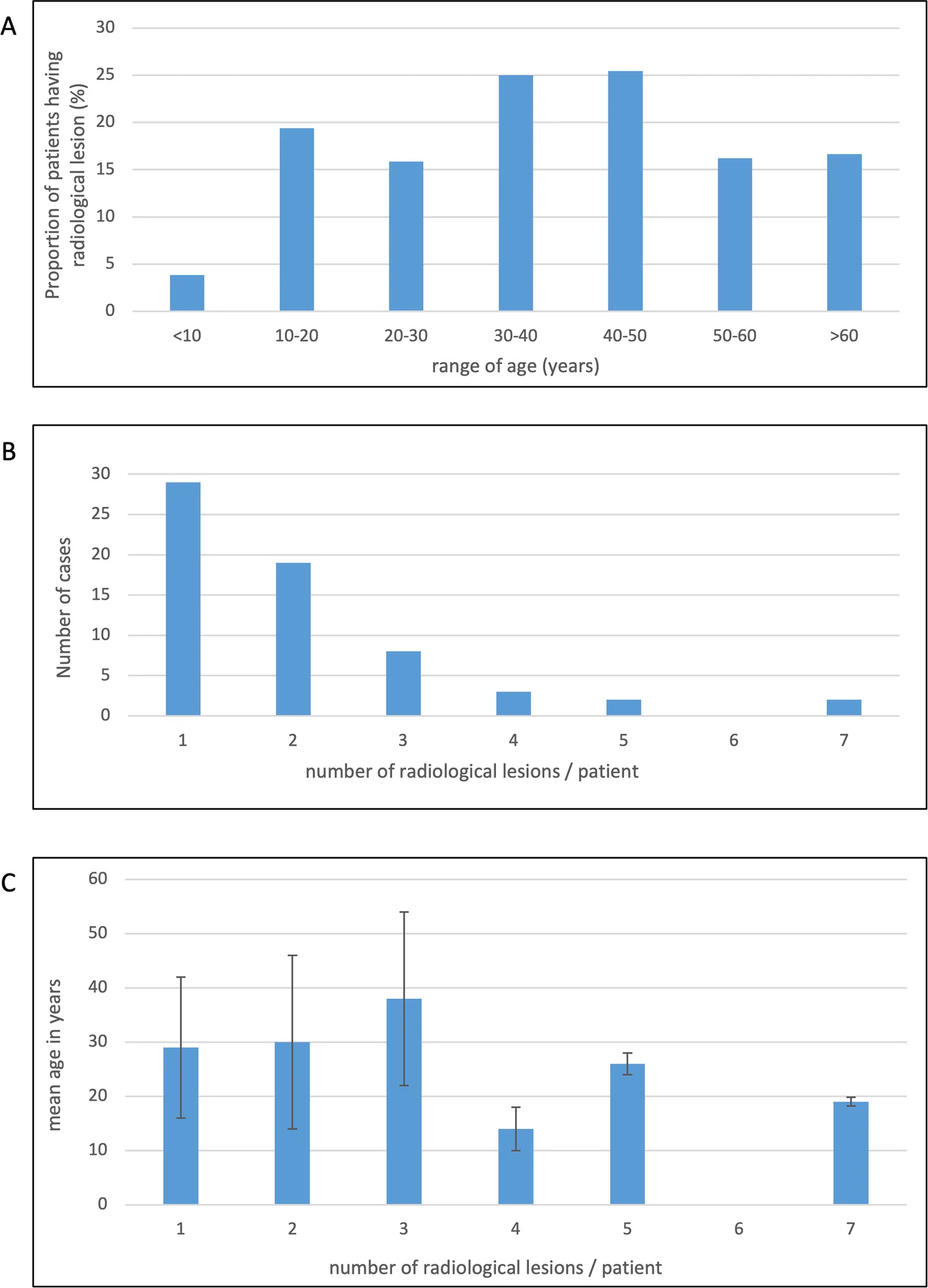
Frequency and distribution of the radiological nonossifying fibroma-like lesions. (a) Frequency in various age-range. (b) Number of the lesions per patient. (c) Mean age of patients with different number of lesions. Bars indicate the SD.

Patients were grouped into ten-year age intervals. The proportion of patients having a bone lesion in each age group was calculated and illustrated in Figure 1. Among patients with lesions, counts of those with various numbers of lesions are also shown in Figure 1.

For each of the 92 cases with MRI available from two different dates, volumes of the two measurements were given and connected with a line (Fig. 4). In addition, annual changes of the volume of the lesions in each patient per year were calculated as: ((volume at follow-up MRI)–(volume at initial MRI))/(years between the two MRIs). This change was plotted against age at the initial MRI.

Data evaluation was descriptive, and no statistical test was performed.

## Results

**Fig. 2:**
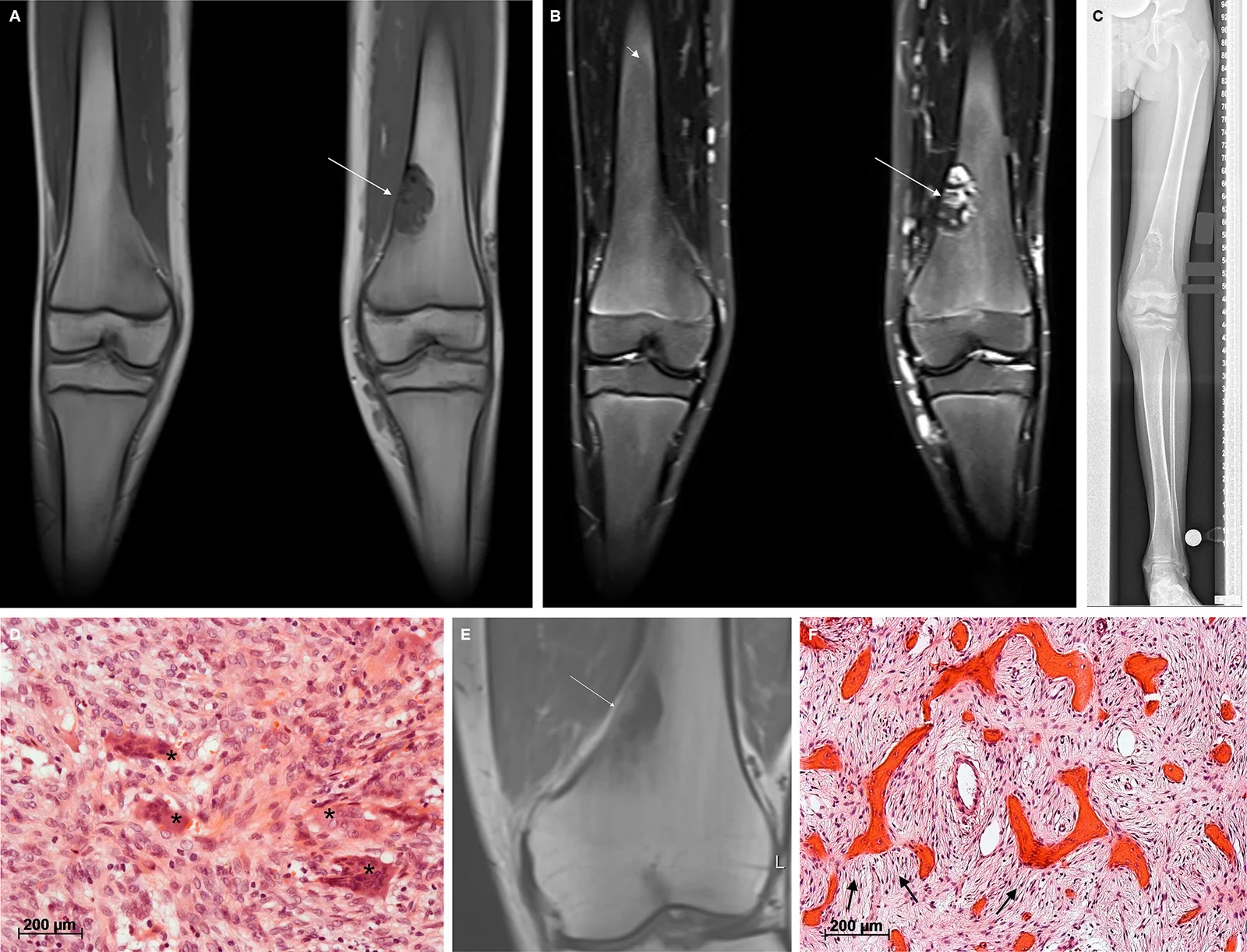
Radiological nonossifying fibroma-like lesions in 13- and 43-year-old female patients. (a, e) T1-weighted MRI; (b) proton-density-suppressed MRI; and (c) radiograph. The white arrows point to the radiological lesions. (d) Haematoxylin-eosin-stained lesion in a 13-year-old female patient showing bland spindle-shaped cells with ovoid nuclei without any atypia, arranged in a storiform pattern, scattered osteoclast-type giant cells (*) and delicate haemosiderin deposits, characteristic of nonossifying fibroma. (e) T1-weighted MRI distal femur of a 43-year-old female patient. (f) Haematoxylin-eosin-stained lesion from a 43-year-old patient showing fibro-osseous tumour of woven bone with bland fibroblastic spindle cells. Prominent activated osteoblasts do not line the irregular trabeculae, and collagenous Sharpey-like fibres arise at right angles from the bone (black arrows), a typical feature of fibrous dysplasia.

A total of 333 patients diagnosed with NF1 and MRI scans were included in this retrospective study. In all, 127 NOF-like lesions were found in 63 patients (19%), 57% being male and 43% being female patients. The lesions were clearly visible in T1-weighted MRI, proton-density-suppressed MRI, and also in radiographs (Figure 2).

Patients’ age ranged from aged six to 70 years. The frequency of the radiological NOF-like lesions varies between age-groups. A peak was seen between aged 30 and 50 years. In these two decades, every fourth patient displayed at least one NOF-like lesion. However, no clear trend of decrease or increase of the frequency with age is visible. The majority of the patients had one lesion, followed by patients who had two, three, and four, and so on. The maximal number of bony lesions per patient was seven. Again, no trend of decrease nor increase of number of lesions per patient with age was visible (Figure 1).

**Fig. 3:**
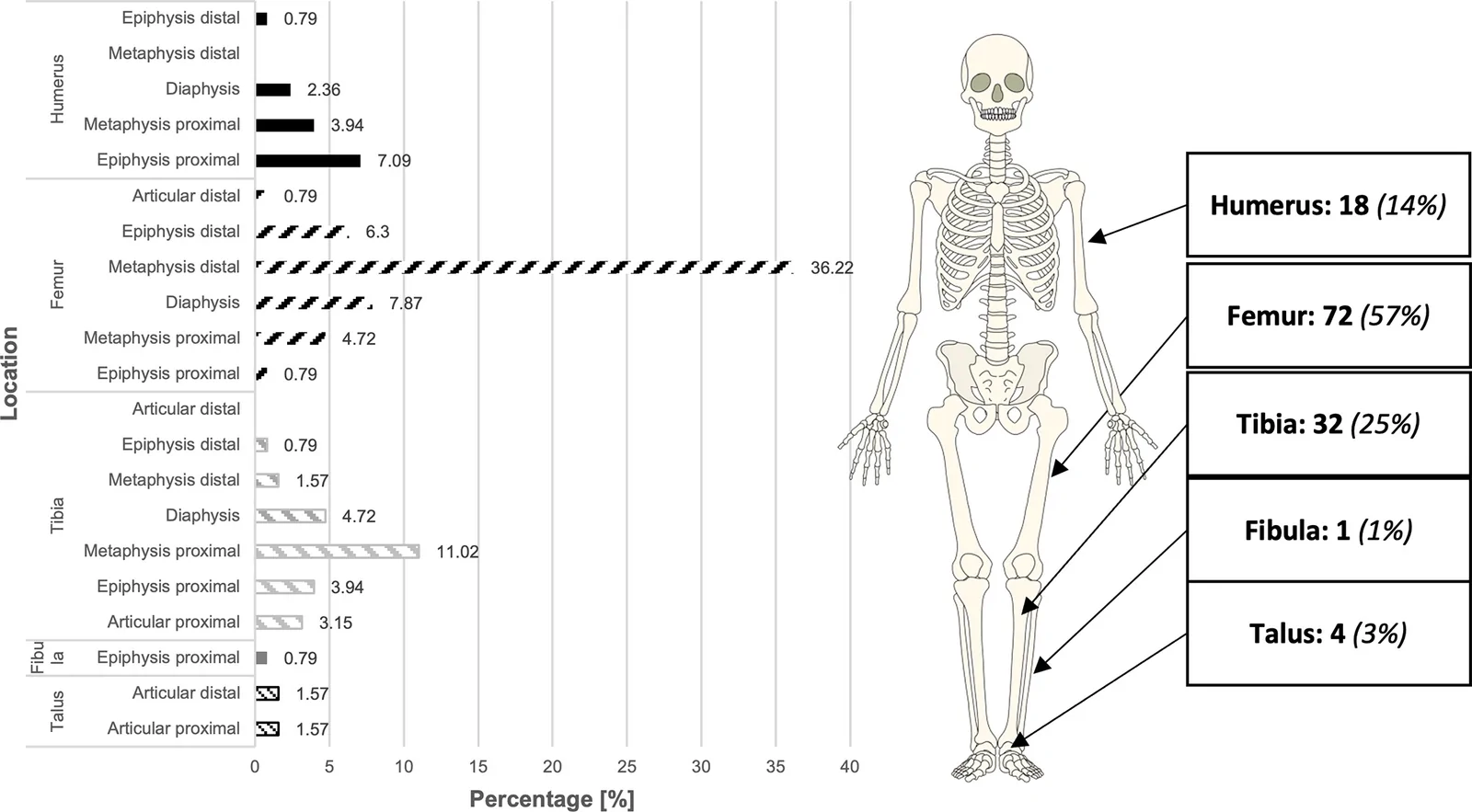
Distribution of the radiological nonossifying fibroma-like lesions in various body regions.

Nearly 90% of the NOF-like lesions are in the lower limb (Figure 3). The majority (58%) of lesions were located in the metaphyses of the affected long bones.

### Radiological characteristics of the bone lesions

Typically, the NOF-like lesions on the MRI scans are eccentric in the affected bone section with variable distance to the (former) growth plate (Figure 2). They are polycyclic in shape, have a sclerotic border that varies in thickness, sometimes protruding into the cortex yet without any periosteal reaction. High or intermediate T2 signals with concomitant low 1 signals were observed, but also lesions with low signal intensity on all sequences. Contrast enhancement was variable. None of the NOF-like lesions showed signs of soft-tissue involvement, severe cortical destruction, or periosteal reactions. Some of these NOF-like lesions included minor osteolytic areas (e.g. metaphyseal or diaphyseal central osteolysis, focal penetration of the cortical bone). Follow-up MRI imaging revealed that these NOF-like lesions were regressive sclerotic rims and the focal cortical destruction healed with time.

### Clinical symptoms

None of the 63 patients with radiological NOF-like lesions had any acute or chronic associated clinical symptoms. Retrospective re-evaluation of their medical records identified ten patients (16%) with potentially associated symptoms. Five patients reported transitional pain of the knee region with NOF-like lesions in the proximity. In all five cases, the pain disappeared spontaneously without specific treatment. Two other patients reported transient symptoms in the shoulder joint and another two patients in the hip joint, all with a radiological lesion in the proximity. No intervention was needed as symptoms disappeared spontaneously. The tenth patient had a tibial fracture with a radiological NOF-like lesion in the proximity, in a motor vehicle accident. It was not a pathological fracture through the lesion. It healed well and no further symptoms were documented thereafter in the follow-up.

### Histology of two NOF-like lesions

Two female patients (aged 13 and 43 years) and their treating physicians were concerned about the radiological NOF-like lesions. Therefore, biopsies were taken for further examination. Histopathology revealed in one case a nonossifying fibroma and an in the other case a fibrous dysplasia with no correlation to a neurogenic differentiated bone tumour or soft-tissue tumour in either case (Figure 2).

### Follow-up

**Fig. 4:**
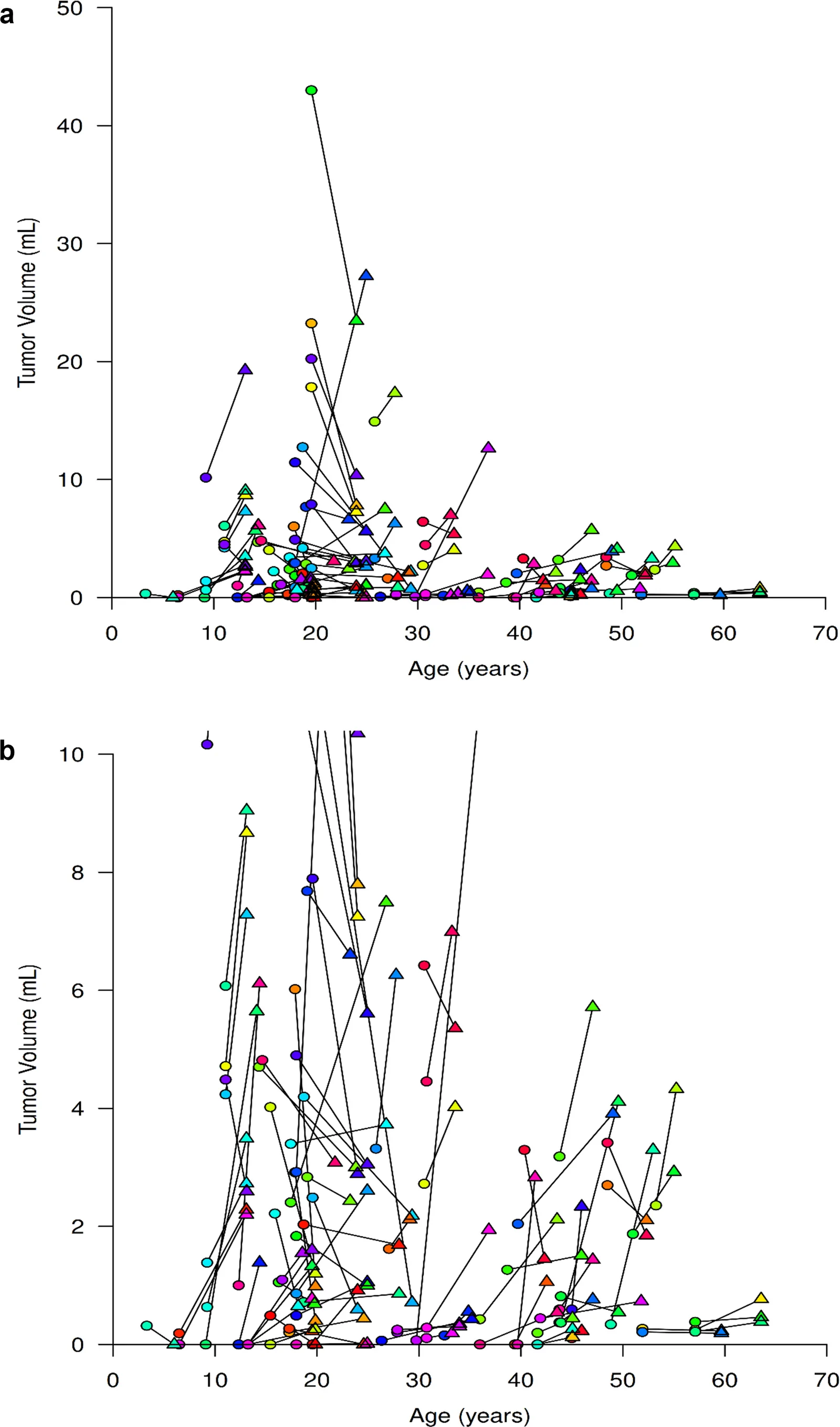
Volumes of the nonossifying fibroma-like lesion at two sequential MRIs were plotted against the ages (years) of the patient at the first and follow-up MRI. Each patient has a specific colour. Circles and triangles represent the volume of the lesion at the initial and the follow-up MRI, respectively. a) Full scale. b) Volume-scale was zoomed in to 0 ml to 10 ml to see more details of the majority of cases.

**Fig. 5:**
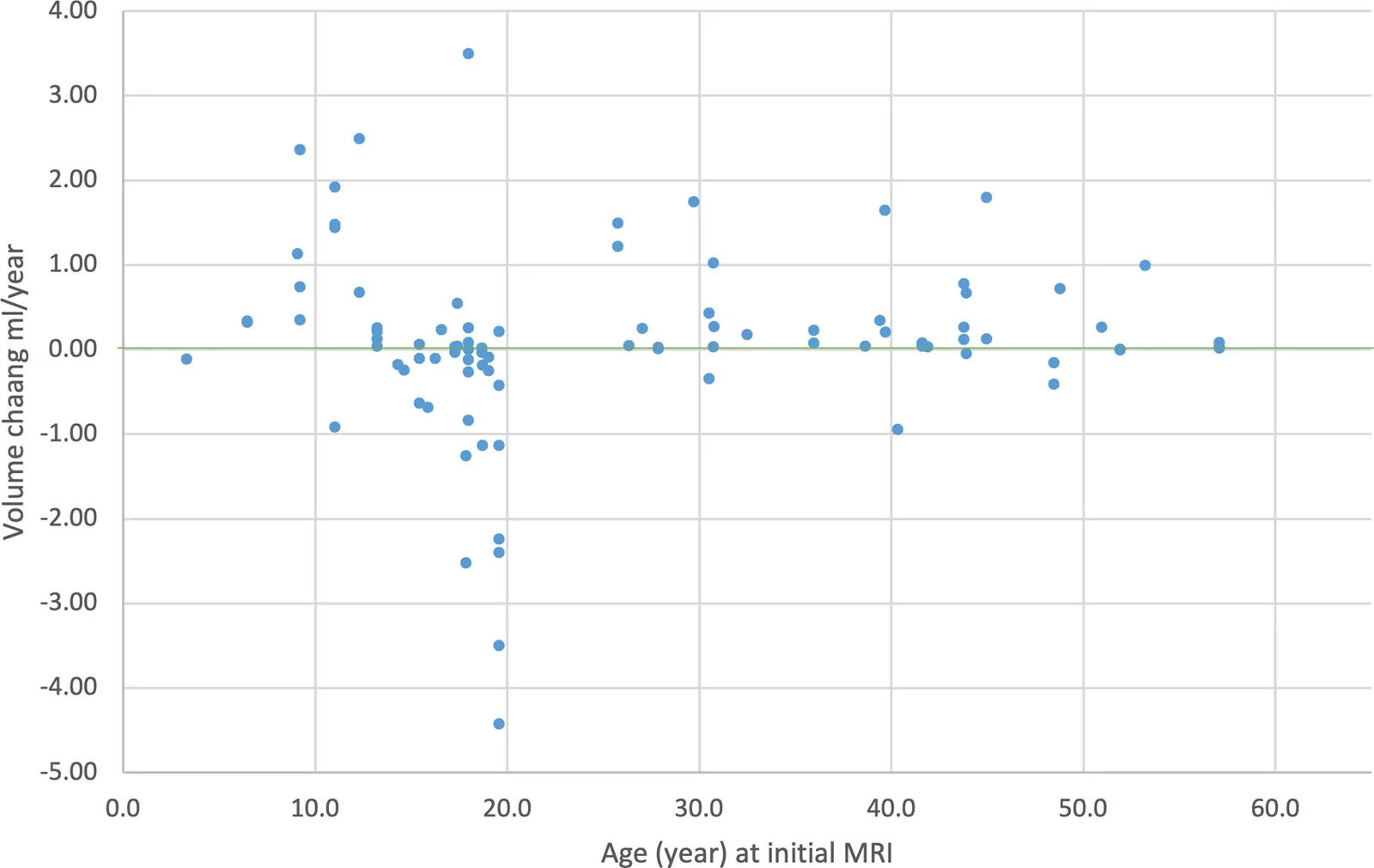
Volume change of the nonossifying fibroma-like lesions per year was calculated as: ((volume at follow-up MRI)–(volume at initial MRI))/(years between the two MRIs), and plotted against age (years) at the initial MRI.

Follow-up MRI of 92 of the 127 lesions with one to 10.6 years (mean five years (SD 2.5)) between initial and latest MRI revealed both increases and decreases in volume (Figure 4 and Figure 5). Increase in volume was observed in a majority of the cases (Figure 4). Although around the age of 20 years, there seem to be more cases with a decrease in volume (Figure 5), there was no consistent pattern of volume change over the entire cohort when looking at the whole age range.

## Discussion

Here, we describe the prevalence of MRI-detectable NOF-like lesions in a unique, large patient population of NF1 patients. Of the 333 NF1 patients, we identified 63 patients with 127 NOF-like lesions. None of these patients had persistent NOF-like lesion-associated symptoms, and none required specific treatment of the bony lesions

To our knowledge, this patient population represents by far the largest cohort of NF1 patients in a single centre with sequential MRI studies that has been described in the literature. Therefore, it is uniquely suited for studying the prevalence and clinical importance of skeletal lesions, which are often coincidental findings. This is relevant, as in the literature, NOF-like lesions in NF1 patients have been reported to be associated with clinical symptoms.^15^ Therefore, diagnosis of these NOF-like lesions often causes considerable uncertainty among physicians and affected patients as how to treat these lesions. Literature on the natural history, prevalence, and clinical relevance of NOF-like lesions in NF1 patients is scarce and in part contradictory.^16,17^ Here, we aimed to describe the epidemiology and clinical relevance of MRI-detectable NOF-like lesions in NF1 patients in a unique, large patient cohort of 333 patients with long-term MRI (interval one to 11 years, mean five years (SD 2.5) and clinical follow-up data.

In a series of 14 cases with native radiograph imaging, a prevalence of 36% has been described,^18^ which is much higher than the 19% we found in the present study. The difference is likely due to sampling error, given the much larger size of our patient population (n = 333) compared with the small group (n = 14) examined in the previously published case series.^18^ In contrast, an analysis of 192 NF1 patients with plane radiograph in the 1960s did not reveal a single NOF-like lesion.^19^ Based on our current data, we would expect MRI-detectable NOF-like lesions in 15% to 20% of 192 patients, corresponding to about 33 patients with NOF-like lesions. This discrepancy may be explained by the fact that in the 1960s, documentation of skeletal lesions was done only by native radiographs and not MRI, while MRI is a much more sensitive method for the detection of small NOF-like lesions.

In the present study, the location of these lesions within the respective bones was mostly eccentric with variable distances from the (former) growth plate and was found around the knee in more than 80%, in particular in the metaphysis of the distal femur. This is comparable with previous studies, who identified the NOF-like lesions in the distal metaphysis of the femur or the proximal metaphysis of the tibia. Additionally, the cortices can expand with increased size on radiographs and on MR-imaging the lesions can have a ‘soap bubble’ appearance.^16-18^ Previous papers warn for potential stress-type fractures in cases of cortical thinning. Here, we demonstrate that the majority of the NOF-like lesions are not associated with clinical symptoms. Few patients feature temporary symptoms of the knee in proximity to bony lesions, all of which have resolved spontaneously. Thus, we found no evidence for a causal relationship between the temporary symptoms and the bony lesions, and conclude that it remains questionable whether the bony lesions do ever become symptomatic. No stress-type fractures were identified in these particular cases with cortical thinning in our cohort. Although histology was obtained in only two patients of the 63 individuals with NOF-like lesions, we think that these can be regarded as representative, because they were taken from lesions with typical radiological appearance in NF1 patients. The MRI appearance of these NOF-like lesions is consistent, but the scans cannot differ between NOF and fibrous dysplasia. The obtained biopsies and the histological diagnoses of nonossifying fibroma and fibrous dysplasia are consistent with the undefined radiological appearance of a NOF-like lesion. Therefore, histopathology is required to clarify the definitive diagnosis. Nonetheless, biopsies are not routinely required, because there is no clinical consequence as long as lesions are asymptomatic.

The prevalence of roughly 20% of NOF-like lesions in NF1 patients is interesting as it may indicate an effect of the genetic defect. However, since we do not compare the present findings with whole-body MRI data from a gender- and age-matched non-NF1 control group, we do not know with certainty whether the prevalence of nearly 20% NOF-like lesions in this large NF1 population is NF1-specific or would be observed in an equally asymptomatic age-matched control group as well.

However, for the central conclusion from the present study, it is irrelevant whether such lesions would also be found in a control group. The major clinical conclusion from this study is that patients and families affected with NF1 do not have to worry about radiological NOF-like lesions. As long as the lesions are asymptomatic, they do not require treatment nor imaging follow-up. And if they are symptomatic, clinical monitoring is usually enough until symptoms disappear. In the possible rare event of a symptomatic lesion that increases in size, further diagnostic workup by biopsy is recommended. However, the main lesson we have learned from the present study is that bony lesions in NF-1 patients are usually asymptomatic even if they grow in size. If uncertainty remains in cases of asymptomatic but growing lesions, a simple radiograph follow-up after a year should be performed to reassure patients and their families; however, continuous routine radiological follow-up is not required as long as NOF-like lesions remain asymptomatic.

It is well known that nonossifying fibromas are frequent in children but rare in adults.^20,21^ Interestingly, in our cohort, the frequency of the NOF-like lesions was highest in the age group of 30 to 50 years. This observation may indicate that these lesions with typical radiological appearance of NOF, should rather be regarded as NOF-like lesions, because their biology appears to differ slightly from what we know as typical NOF. It is conceivable that the underlying genetic defect in NF1 patients affects a shift of the age peak of NOF-like lesions to the right. The pattern of volume change from follow-up data also suggests a NF1-associated shift of NOF-like lesions from children to adults in NF1. This observation may indicate a delayed bone maturation in NF1 patients, but the potential underlying mechanism of this association and shift currently remains unknown.

The retrospective nature of the study is a limitation. It cannot be ruled out that there is a certain degree of under-reporting and under-documentation of clinical symptoms. However, since this is a unique setting for NF1 patients, with strong patient adherence to the unit and a very systematic and thorough routine clinical history and follow-up exam by the specialized health care professional team at fixed intervals, we consider it unlikely that relevant information on musculoskeletal symptoms is missing to a meaningful extent. Whenever musculoskeletal symptoms appeared in any of these patients, a consultation with the orthopaedic department was routinely requested and documented.

In conclusion, NOF-like lesions are frequent on whole-body MRI scans of NF1 patients with a prevalence of roughly 20% over all age groups. These lesions normally do not cause clinical symptoms and do not require therapeutic intervention.

**Take home message**

- Nonossifying fibroma (NOF)-like lesions are frequent on whole-body MRI scans of neurofibromatosis type 1 (NF1) patients.

- However, there is no association with clinical symptoms. These NOF-like lesions should be recognized as “no-touch” lesions.

## Data Availability

The datasets generated and analyzed in the current study are not publicly available due to data protection regulations.

## References

[1] Lammert M Friedman JM Kluwe L Mautner VF Prevalence of neurofibromatosis 1 in German children at elementary school enrollment Arch Dermatol 2005 141 1 71 74 10.1001/archderm.141.1.71 15655144

[2] Gutmann DH Ferner RE Listernick RH Korf BR Wolters PL Johnson KJ Neurofibromatosis type 1 Nat Rev Dis Primers 2017 3 17004 10.1038/nrdp.2017.4 28230061

[3] Friedrich RE Schmelzle R Hartmann M Fünsterer C Mautner V-F Resection of small plexiform neurofibromas in neurofibromatosis type 1 children World J Surg Oncol 2005 3 1 6 10.1186/1477-7819-3-6 15683544 PMC549083

[4] Salamon J Mautner VF Adam G Derlin T Multimodal imaging in neurofibromatosis type 1-associated nerve sheath tumors Rofo 2015 187 12 1084 1092 10.1055/s-0035-1553505 26333104

[5] Friedrich RE Kluwe L Fünsterer C Mautner VF Malignant peripheral nerve sheath tumors (MPNST) in neurofibromatosis type 1 (NF1): diagnostic findings on magnetic resonance images and mutation analysis of the NF1 gene Anticancer Res 2005 25 3A 1699 1702 16033085

[6] Mautner V-F Asuagbor FA Dombi E et al. Assessment of benign tumor burden by whole-body MRI in patients with neurofibromatosis 1 Neuro Oncol 2008 10 4 593 598 10.1215/15228517-2008-011 18559970 PMC2666233

[7] Nguyen R Jett K Harris GJ Cai W Friedman JM Mautner VF Benign whole body tumor volume is a risk factor for malignant peripheral nerve sheath tumors in neurofibromatosis type 1 J Neurooncol 2014 116 2 307 313 10.1007/s11060-013-1293-1 24166582

[8] Tsirikos AI Saifuddin A Noordeen MH Spinal deformity in neurofibromatosis type-1: diagnosis and treatment Eur Spine J 2005 14 5 427 439 10.1007/s00586-004-0829-7 15712001 PMC3454658

[9] Van Royen K Brems H Legius E Lammens J Laumen A Prevalence of neurofibromatosis type 1 in congenital pseudarthrosis of the tibia Eur J Pediatr 2016 175 9 1193 1198 10.1007/s00431-016-2757-z 27519821

[10] Heervä E Leinonen P Kuorilehto T et al. Neurofibromatosis 1-related osteopenia often progresses to osteoporosis in 12 years Calcif Tissue Int 2013 92 1 23 27 10.1007/s00223-012-9661-y 23108390

[11] World Medical Association Declaration of Helsinki: ethical principles for medical research involving human subjects JAMA 2013 310 20 2191 2194 10.1001/jama.2013.281053 24141714

[12] Heverhagen JT Hahn HK Wegmann M et al. Volumetric analysis of mice lungs in a clinical magnetic resonance imaging scanner MAGMA 2004 17 2 80 85 10.1007/s10334-004-0053-9 15480944

[13] Feusner JH Beckwith JB D’Angio GJ Clear cell sarcoma of the kidney: accuracy of imaging methods for detecting bone metastases. Report from the National Wilms’ tumor study Med Pediatr Oncol 1990 18 3 225 227 10.1002/mpo.2950180311 2158615

[14] Meinberg EG Agel J Roberts CS Karam MD Kellam JF Fracture and dislocation classification compendium 2018 J Orthop Trauma 2018 32 Suppl 1 S1 S170 10.1097/BOT.0000000000001063 29256945

[15] Diallo ID Iraqi Houssaini Z Tantaoui M et al. Bone manifestations of neurofibromatosis type 1 Glob Pediatr Health 2022 9 2333794X221101771 10.1177/2333794X221101771 35664048 PMC9160888

[16] Mankin HJ Trahan CA Fondren G Mankin CJ Non-ossifying fibroma, fibrous cortical defect and Jaffe-Campanacci syndrome: a biologic and clinical review Chir Organi Mov 2009 93 1 1 7 10.1007/s12306-009-0016-4 19711155

[17] Remotti F Feldman F Nonneoplastic lesions that simulate primary tumors of bone Arch Pathol Lab Med 2012 136 7 772 788 10.5858/arpa.2011-0557-RA 22742550

[18] Mandell GA Dalinka MK Coleman BG Fibrous lesions in the lower extremities in neurofibromatosis AJR Am J Roentgenol 1979 133 6 1135 1138 10.2214/ajr.133.6.1135 116506

[19] Hunt JC Pugh DG Skeletal lesions in neurofibromatosis Radiology 1961 76 1 20 10.1148/76.1.1 13716855

[20] Breden S Stephan M Hinterwimmer F et al. Pediatric bone tumors: location and age distribution of 420 cases Diagnostics (Basel) 2024 14 22 2513 10.3390/diagnostics14222513 39594179 PMC11593068

[21] De Salvo S Pavone V Coco S Dell’Agli E Blatti C Testa G Benign bone tumors: an overview of what we know today J Clin Med 2022 11 3 699 10.3390/jcm11030699 35160146 PMC8836463

